# The Prognostic value of the Fibrinogen to pre-albumin ratio in malignant tumors of the digestive system: a systematic review and meta-analysis

**DOI:** 10.1186/s12935-022-02445-w

**Published:** 2022-01-15

**Authors:** Baibei Li, Huachu Deng, Ziyan Zhou, Bo Tang

**Affiliations:** 1 Department of Hepatobiliary Surgery, The First Affiliated Hospital of Guangxi Medical University, No 6 Shuangyong Road, Nanning, 530021 Guangxi People’s Republic of China; 2 Department of Gastrointestinal Surgery, the First Affiliated Hospital of Guangxi Medical University, Nanning, 530021 Guangxi People’s Republic of China; 3 Department of Radiation Oncology, the First Affiliated Hospital of Guangxi Medical University, Nanning, 530021 Guangxi People’s Republic of China

**Keywords:** Fibrinogen to pre-albumin ratio, Prognosis, Digestive cancers

## Abstract

**Background:**

In recent years, the Fibrinogen to pre-albumin ratio (FPR) has been reported in many studies to be significantly associated with the prognosis of various cancers. This systematic review and meta-analysis aimed to investigate the prognostic value of FPR in malignant tumors of the digestive system based on available evidence.

**Methods:**

The relevant articles published before July 1, 2021, were systematically retrieved from electronic databases to evaluate the effect of Fibrinogen to pre-albumin ratio (FPR) on the prognosis of patients with malignant digestive system tumors and calculate the hazard ratio (HR) and the corresponding 95% confidence interval (CI).

**Result:**

Thirteen articles, all from China, including 15 cohort studies and a total of 5116 cases, were included in this study. A high FPR was associated with poor overall survival (HR = 1.88, 95%CI 1.53–2.32, P < 0.001), recurrence-free survival (HR = 2.29, 95%CI 1.91–2.76, P < 0.001), progression-free survival (HR = 1.96, 95%CI: 1.33–2.90, P = 0.001), complications (HR = 1.78, 95%CI: 1.06–3.00, P = 0.029), disease-free survival (HR = 1.46, 95%CI: 1.08–1.97, P = 0.013) was significantly associated with cancer-specific survival (HR = 1.44, 95%CI: 1.15–1.79, P = 0.001). Even though intergroup differences were present, FPR was strongly associated with overall and relapse-free survival, and sensitivity analysis suggested that our results were stable.

**Conclusion:**

FPR can be used as a valuable indicator to predict the prognosis of patients with malignant digestive system tumors.

## Introduction

Malignant tumors of the digestive system are among the most common malignancies globally, including colorectal carcinoma, carcinoma of the stomach, hepatic carcinoma, and esophageal carcinoma [1].Most patients are unfortunately diagnosed at advanced cancer stages. According to the clinical staging, early cases of gastrointestinal cancers are usually treated by surgical resection and postoperative adjuvant therapy. For advanced cancers, chemotherapy and radiotherapy are the mainstays of oncologic therapy and can only modestly prolong the survival of patients. Due to intratumoral heterogeneity, malignant tumors are often resistant to chemotherapeutic drugs, leading to disease recurrence and a poor overall patient prognosis. Few oncologic treatment options are available in advanced diseases; accordingly, it is essential to explore new biomarkers such as the Fibrinogen to pre-albumin ratio (FPR) to help clinicians during prognostic evaluation and assist in decision making.

Fibrinogen is a 340 KDa hepatocyte-produced glycoprotein that can be converted to fibrin by activated thrombin. It modulates the coagulation and thrombosis process and plays an important role in hemostasis, cell attachment, and systemic inflammatory reactions [2].High Fibrinogen is an important risk factor for various thrombotic diseases and is also considered a marker of blood hypercoagulability in clinical practice, which is closely related to the occurrence and prognosis of various cardiovascular diseases [3]. Fibrinogen is also acknowledged as an acute-phase protein produced by the liver and is significantly elevated during an infection or inflammatory disease. Thus, it can also be used as a marker to mirror the inflammation level in an organism. Interestingly, malignant tumor cells can partially express Fibrinogen [4].Fibrinogen has also been associated with vascular endothelial cell growth factor (VEGF) and fibroblast growth factor 2 (FGF-2) and can promote tumor cell adhesion, proliferation and migration [5].Elevated serum fibrinogen levels are usually associated with poor prognosis of human cancers [6, 7]. Pre-albumin, produced primarily in the liver, is a transportation protein found primarily in the blood. Its main functions are to bind and transport thyroid hormones and vitamin A [8]. Furthermore, serum pre-albumin is an important biomarker for assessing the nutritional status of cancer patients [9]. Reduced pre-albumin levels have also been documented in cancer patients and are associated with a poor prognosis.

Even though pre-albumin and Fibrinogen are all widely acknowledged to be associated with tumor prognosis, the Fibrinogen to pre-albumin ratio (FPR) has rarely been reported to evaluate the prognosis of patients with digestive tumors. At present, only one meta-analysis mentioned the correlation between FPR and OS of cancer patients, which proved that high FPR was associated with poor OS in cancer patients, but there was no independent report on the relationship between FPR and malignant digestive system tumors, and there was no report on the relationship between FPR and recurrence-free survival [10]. Therefore, the aim of this study was to conduct a systematic review and meta-analysis of available evidence to assess the prognostic value of FPR in malignant digestive system tumors.

## Methods

### Search strategy and selection criteria

This systematic review followed the Preferred Reporting Items for Systematic Reviews and Meta-Analyses (PRISMA) guidelines [11].In this regard, the databases of the Cochrane Library, Embase, PubMed, Google Scholar, Baidu Scholar, CNKI and VIP were searched for relevant articles published not later than July 1, 2021. In addition, we also mine studies that meet the inclusion criteria from TCGA and GEO databases. The complete search strategy is as follows: ((“fibrinogen to pre-albumin ratio” or "fibrinogen” or "pre-albumin” or "FPR”) AND ("colorectal cancer” or "gastric cancer” or "liver cancer” or "esophageal cancer” or “cancer” or “carcinoma”)). Wildcards and Boolean operators were used to perform a comprehensive search.

The eligibility criteria were defined according to the Population, Intervention, Comparison, Outcome and Study Design (PICOS) strategy. Patients with digestive tumors were identified as "Population" at the time of retrieval; high FPR and low FPR represented "Intervention" and "Comparison" respectively; overall survival (OS), recurrence-free survival (RFS), cancer-specific survival (CSS), disease-free survival (DFS), progression-free survival (PFS) and complication were the "Outcome" of this study; "Study design" choice was retrospective and prospective research, and case reports, letters, reviews, editorials and comments were ruled out. If there were duplicates, the most recently published record would be included.

### Data extraction

Duplicates were removed using EndNote (version X8), and eligible studies were entered into a database built by EpiData (version 3.0). Two reviewers independently extracted the required data from the included studies using a special Excel spreadsheet, including author name, publication date, study location, study design (prospective or retrospective research), type of patient, sample size, age composition, gender, primary treatment (surgical and others), cutoff value, methods for choosing FPR cutoff value, outcome, data source (crude data or fitted curve) and duration of follow-up. Outcome indicators included primary (OS and RFS) and secondary outcome indicators (CSS, DFS, PFS and complications). The inclusion of relevant literature and the processing of all data were performed by two independent reviewers (BL and HD). Points of disagreement were reconciled by a discussion with a third reviewer.

### Statistical analysis

All statistical analyses were performed using STATA (version 12). The extracted data was used for calculating hazard ratios (HRs) and 95% CI and used to evaluate the prognostic value of FPR in digestive system malignant tumors. I^2^ statistics were used to quantify the heterogeneity between the included studies and calculated the proportion of variation due to heterogeneity rather than due to chance; I^2^ values ranged from 0 to 100%, with higher values indicating greater heterogeneity. If I^2^ > 50%, the random-effects model was chosen; otherwise, the fixed-effects model was selected. Moreover, subgroup analysis was conducted to explore whether publishing time, methods for choosing FPR cutoff value (ROC or X-tile), cutoff value, types of cancer (colorectal cancer, gallbladder cancer, gastric cancer, hepatocellular cancer or esophageal squamous cell cancer), study design (prospective or retrospective), treatment option (surgical resection or others) and sample capacity had any influence on the results. In addition, sensitivity analysis was used to evaluate the reliability and stability of the results, and Begger's and Egger's tests were used to test whether there was a potential publication bias in the study. P > 0.05 is considered to be free of publication bias, otherwise the trim-and-fill method will be used for reevaluation. All tests were bilateral tests, and P < 0.05 was considered to be statistically significant.

## Results

### Study characteristics

**Fig. 1:**
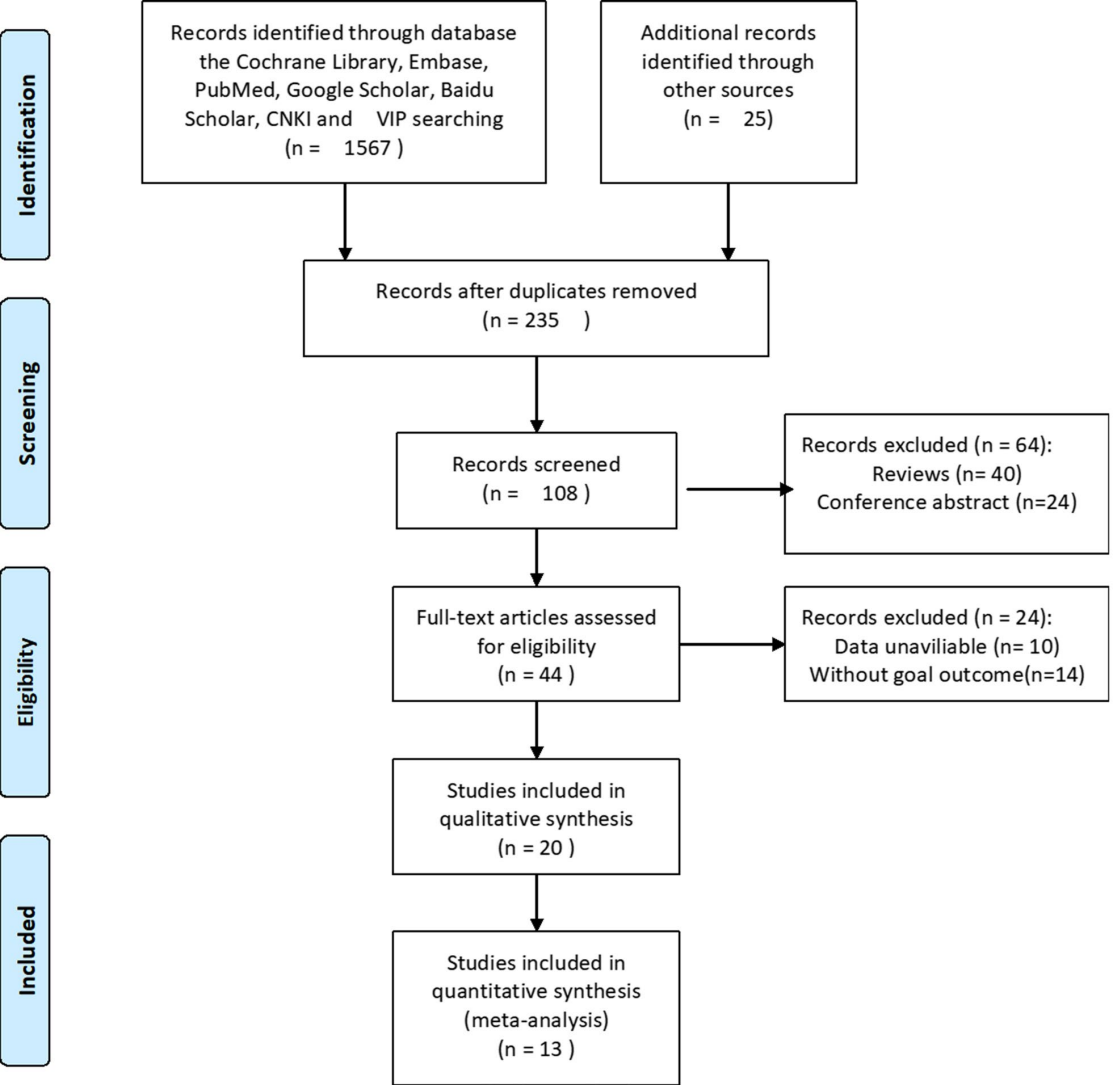
The flow chart of the literature selection

The flowchart documenting retrieval of studies from electronic databases is illustrated in Fig. 1. A total of 13 relevant articles were included in this study, including 15 cohort studies and 5116 patients [12–24]. Unfortunately, we have not found any studies that meet the inclusion criteria from TCGA and GEO databases. Among the included articles, all the studies were conducted in China,7 were retrospective analyses, and 8 were prospective analyses, the year of publication was from 2017 to 2021,the sample size was 42–1014, and the FPR cutoff value was 0.014–31.84.The meta-analysis involved a variety of malignant digestive tumors, including colorectal carcinoma (CRC), carcinoma of the stomach (GC), carcinoma of gall bladder (GBC), hepatic carcinoma (HCC), esophageal squamous cell carcinoma (ESCC) and colorectal mucinous adenocarcinoma (CMA). In addition, ten studies reported OS, four studies reported RFS, two studies reported PFS, and one study reported on postoperative complications, CSS, and DFS. Baseline information for each study is shown in Table 1.

**Table 1:** The characteristics of included studies

Study	Year	Country	Cancer site	Design type	Sample	Gender ratio	Treatment	Outcome	Optimal cut-off for FPR	Follow-up (months)
Liqun Zhang et al	2019	China	CRC	Retrospective	71	44/27	chemotherapy	PFS	18.49 by ROC	Median 6.67 (1.86–27.17)
Jing Zhang et al	2017	China	GC	Retrospective	360	261/99	Surgical resection	OS	12.1 by X-tile	More than 36
Fan Sun et al	2018	China	CRC	Prospective	555	350/205	Surgical resection	OS	18.3 by X-tile	More than 36
Jinghui Du et al	2019	China	GBC	Retrospective	220	122/98	chemotherapy	OS	31.84 by X-tile	More than 36
Lei Zhang et al	2018	China	HCC	Prospective	230	193/37	Surgical resection	RFS,OS	15.6 by X-tile	More than 36
Shuli Tang et al	2020	China	GC	Prospective	273	197/76	Surgical resection	OS	0.0145 by ROC	More than 60
Jifeng Feng et al	2020	China	ESCC	Retrospective	372	327/45	Surgical resection	CSS,OS	0.014 by ROC	More than 60
Yucui Liao et al	2021	China	CMA	Retrospective	42	22/20	chemotherapy	PFS	26.2 by X-tile	More than 36
Hailun Xie et al	2021	China	CRC	Retrospective	584	363/221	Surgical resection	complication, DFS, OS	23.1 by X-tile	More than 60
Hanliang Zhou et al	2020	China	CRC	Prospective	212	133/79	Surgical resection	OS	18.0 by X-tile	More than 36
Qinggen Chen et al	2019	China	CRC	Prospective	77	NA	chemotherapy	OS	22.8 by X-tile	More than 36
Qinggen Chen et al	2019	China	CRC	Prospective	430	NA	mixed	OS	22.8 by X-tile	More than 36
Houqun Ying et al	2021	China	CRC	Prospective	1014	622/392	Surgical resection	RFS	18.3 by X-tile	More than 36
Houqun Ying et al	2021	China	CRC	Prospective	519	348/171	Surgical resection	RFS	18.3 by X-tile	More than 36
Yucui Liao et al	2021	China	CRC	Retrospective	157	91/66	Surgical resection	RFS	19.5 by X-tile	More than 36

### FPR and overall survival

**Fig. 2:**
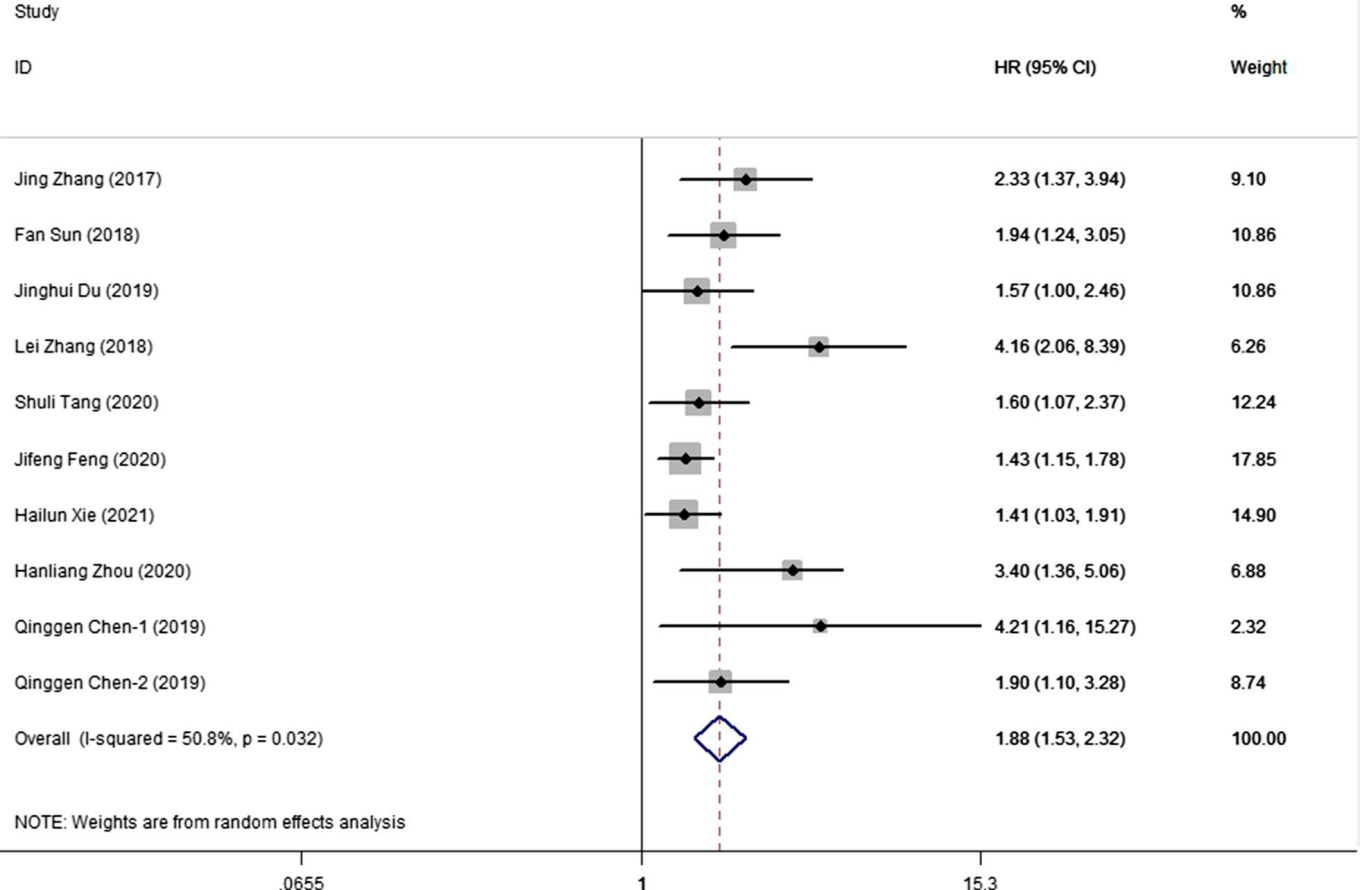
Forest plot for the association between FPR and overall survival

A total of 3313 patients included in 10 cohort studies were analyzed to assess the prognostic significance of FPR levels on OS in malignant digestive tumors. The combined forest plot showed that a high FPR was associated with poor OS (HR = 1.88, 95%CI 1.53–2.32, p < 0.001). Due to the significant heterogeneity (I^2^ = 50.8%, p = 0.032), we used the random effect model (Fig. 2) and performed a hierarchical subgroup analysis by time of publishing, sample size, cutoff value, methods for choosing FPR cutoff value, cancer site, treatment option and designed type (Table 2). High FPR was significantly associated with poor OS, although intergroup differences were present. Moreover, the heterogeneity in some subgroups was eliminated when we stratified according to factors, including "ROC", "sample capacity ≥ 330", "retrospective" and "other treatment options".

**Table 2:** Subgroup Meta-analysis of FPR and OS

Subgroup	No. of cohorts	No. of patients	Pooled HR (95% CI)	P	Heterogeneity *I*^2^ (%) P_h_	
Altogether	10	3313	1.88(1.53,2.32)	< 0.001	50.8	0.032
Publishing time						
< 2020	6	1872	2.10 (1.67,2.64)	< 0.001	25.9	0.24
≥ 2020	4	1441	1.61 (1.25,2.09)	< 0.001	53.0	0.095
Sample capacity						
< 330	5	1012	2.39 (1.55,3.70)	< 0.001	60.9	0.037
≥ 330	5	2301	1.57 (1.35,1.83)	< 0.001	14.3	0.323
Methods for choosing FPR cut-off value						
ROC	2	645	1.47(1.21,1.78)	< 0.001	0.0	0.635
X-tile	8	2668	2.11(1.61,2.77)	< 0.001	50.8	0.047
Cut-off value						
< 18	4	1235	1.96 (1.34,2.89)	0.001	69.8	0.019
≥ 18	6	2078	1.75(1.44,2.12)	< 0.001	39.0	0.146
Study designed type						
Retrospective	4	1536	1.50 (1.28,1.76)	< 0.001	2.3	0.381
Prospective	6	1777	2.32 (1.68,3.19)	< 0.001	43.6	0.115
Treatment option						
Surgical resection	7	2586	1.92 (1.48,2.48)	< 0.001	62.8	0.013
Others	3	727	1.80 (1.29,2.52)	0.001	3.0	0.357
Cancer site						
GC	2	633	1.83(1.33,2.51)	< 0.001	20.8	0.261
CRC	5	1858	2.00(1.42,2.81)	< 0.001	49.5	0.095
GBC	1	220	1.57(1.00,2.46)	0.049	NA	NA
HCC	1	230	4.16(2.06,8.39)	< 0.001	NA	NA
ESCC	1	372	1.43(1.15,1.78)	0.002	NA	NA

### Sensitivity analysis and Publication bias for OS

**Fig. 3:**
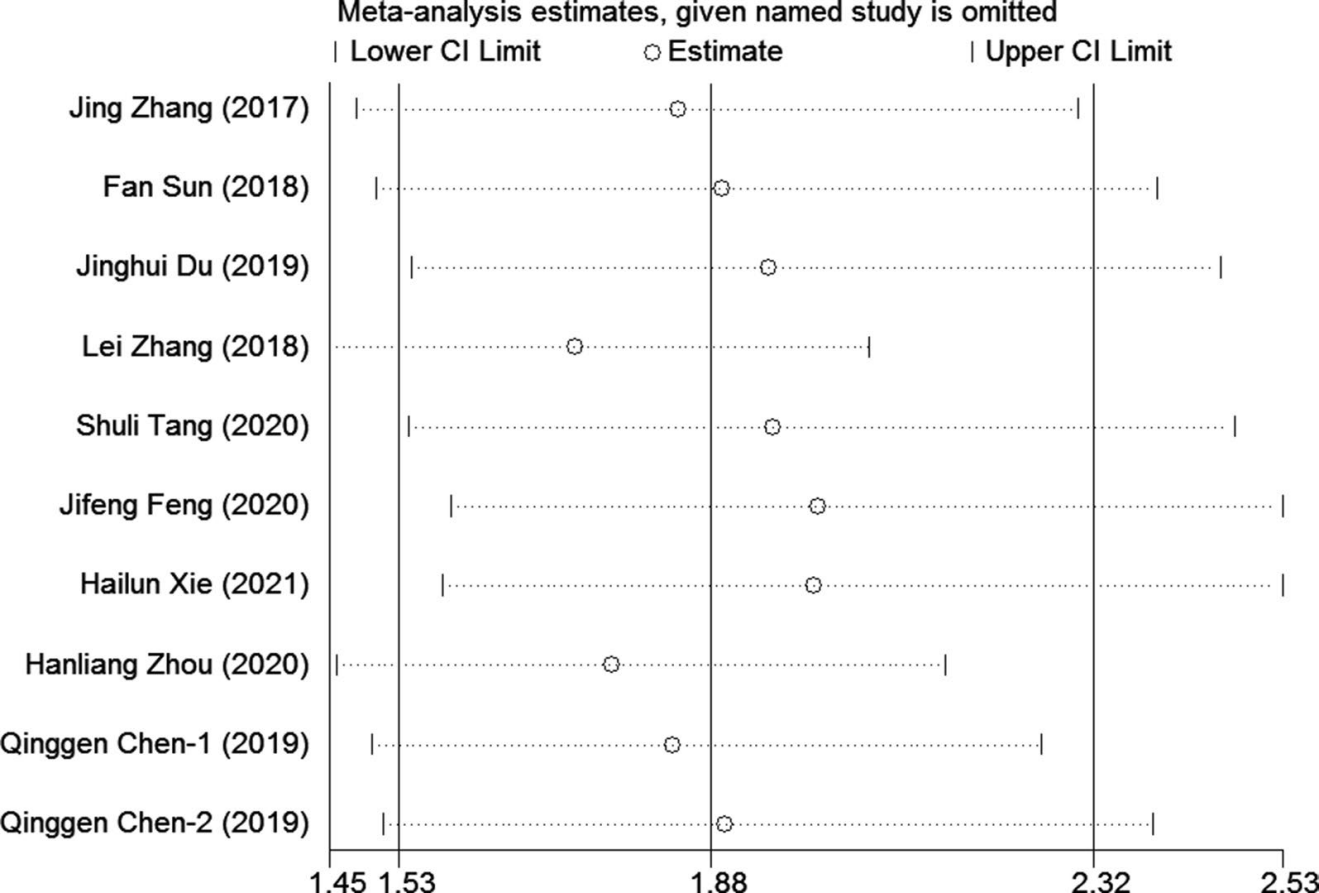
Sensitivity analysis for the association between FPR and OS. OS: overall survival

**Fig. 4:**
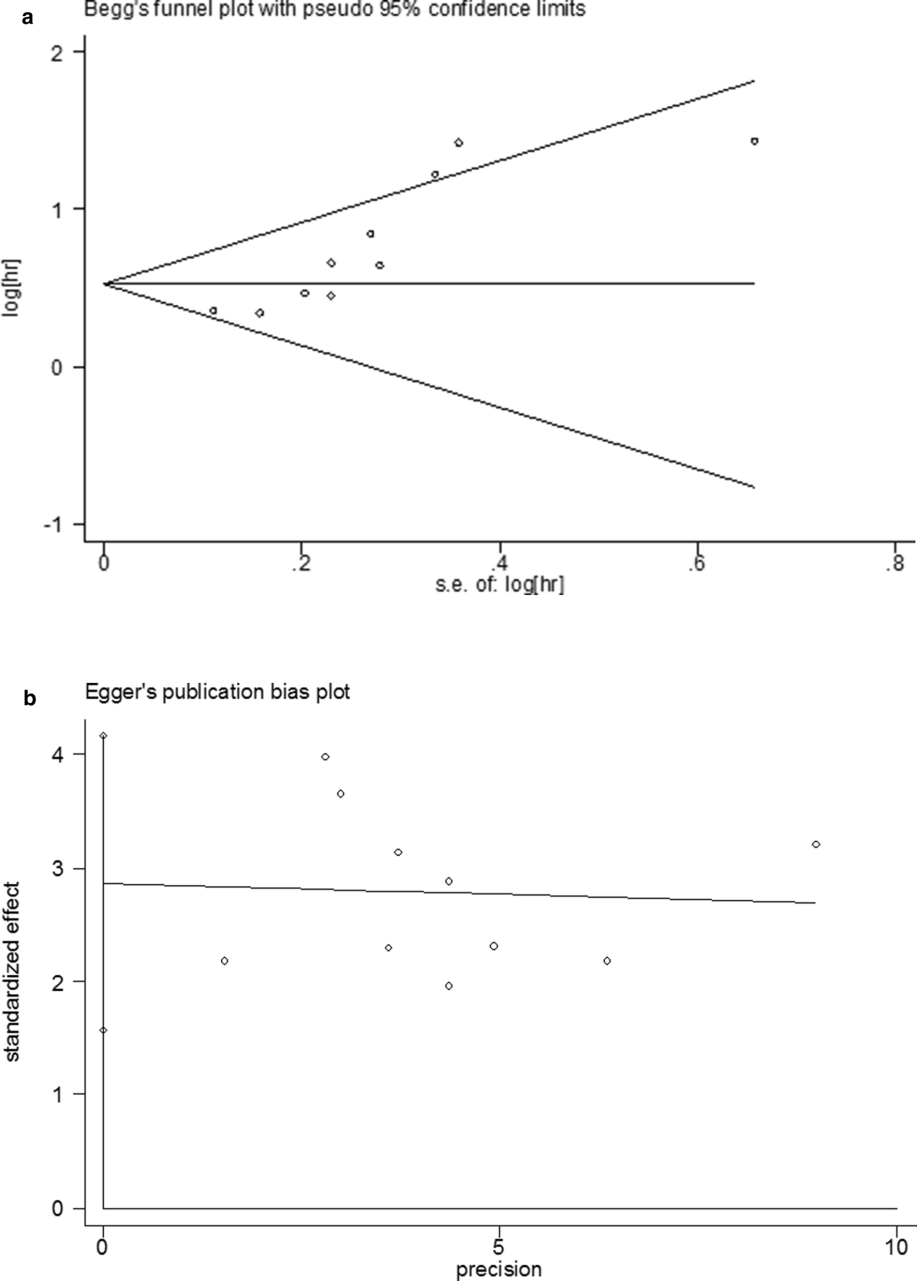

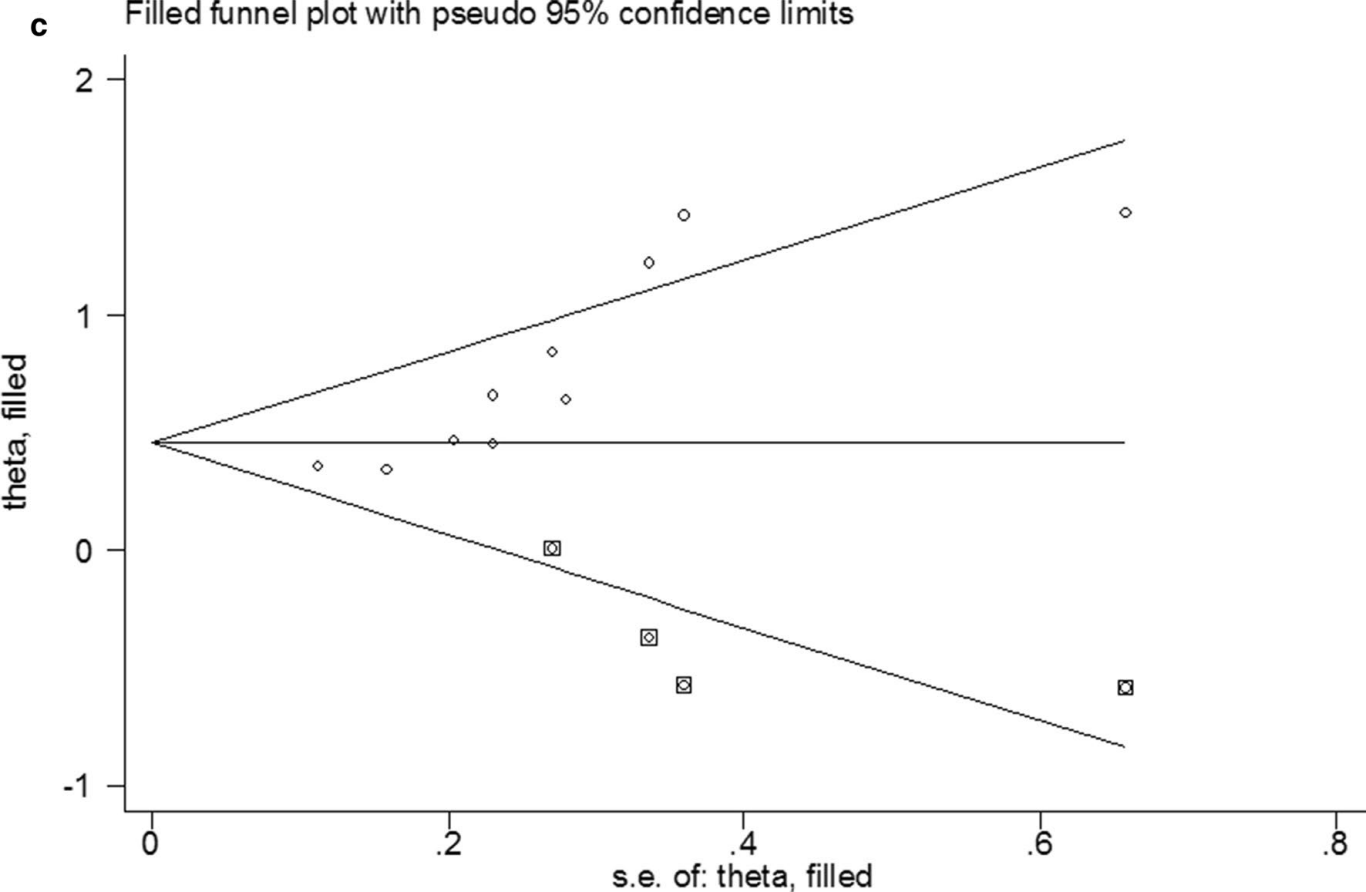
Plots for publication bias test in meta-analysis for overall survival. **a** Begg’s funnel plot; **b** Egger’s publication bias plot; **c** The trim-and-fill methods;

Sensitivity analysis was used to assess the influence of individual studies on the aggregate result, with one inclusion study deleted at a time. The results showed that all of the included studies were close to the centerline, and omitting any of the studies did not change the significant effect of FPR on OS, demonstrating that our results were robust and reliable (Fig. 3) In the present study, Begg's test (p = 0.004) and Egger's test (p = 0.001) were used to investigate publication bias in the included literature, and the funnel plots were asymmetric (p < 0.05), suggesting that there might be publication bias in this study (Fig. 4a, b). Then, we use the trim-and-fill method to identify and correct possible publication biases. A symmetrical funnel diagram was obtained by adding four studies, nonetheless, the corrected HR did not change significantly (HR = 1.572, 95% CI 1.242–1.989, p < 0.001), suggesting that our conclusion is stable (Fig. 4c).

### FPR and recurrence-free survival

**Fig. 5:**
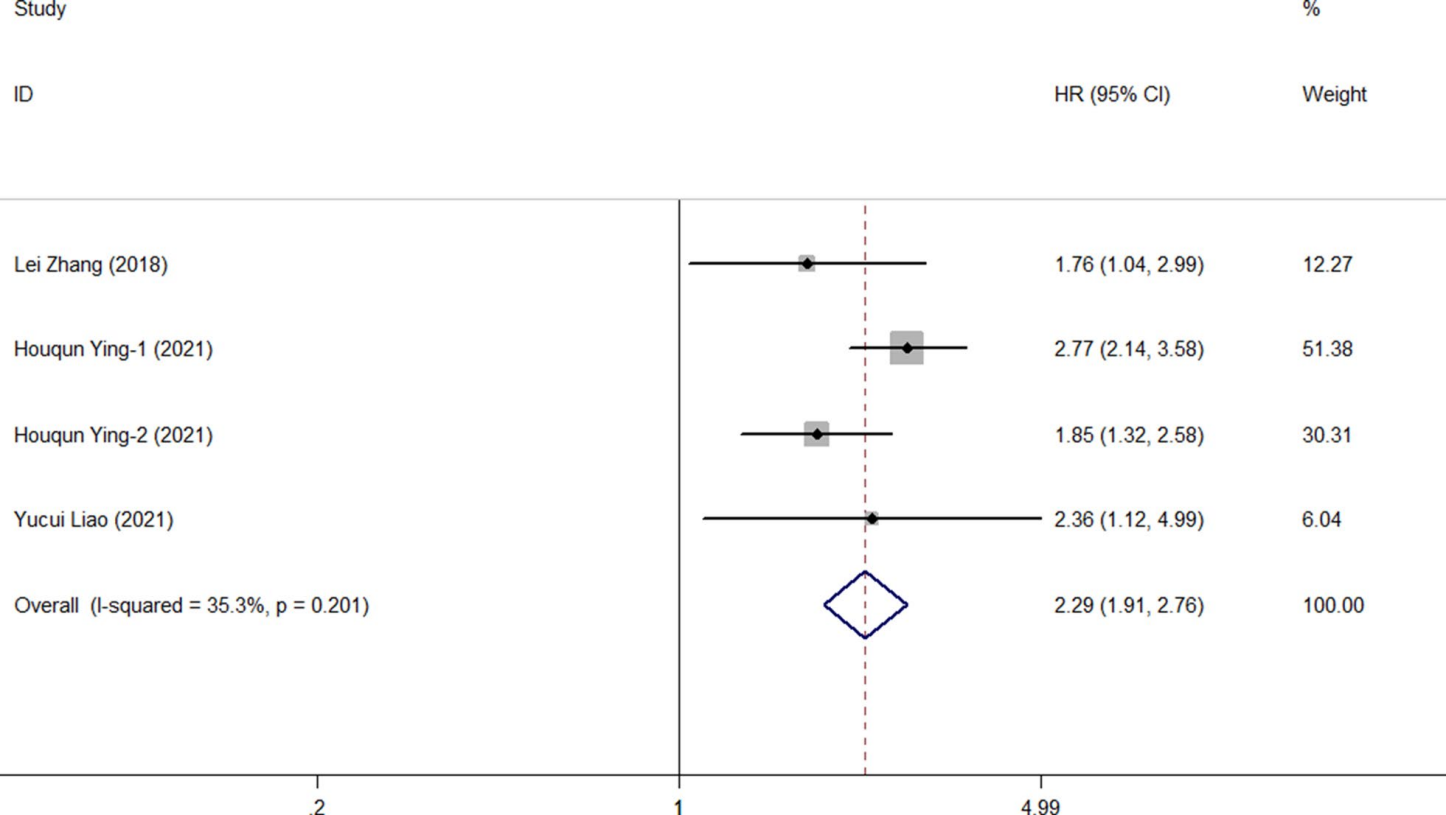
Forest plot for the association between FPR and recurrence-free survival

**Fig. 6:**
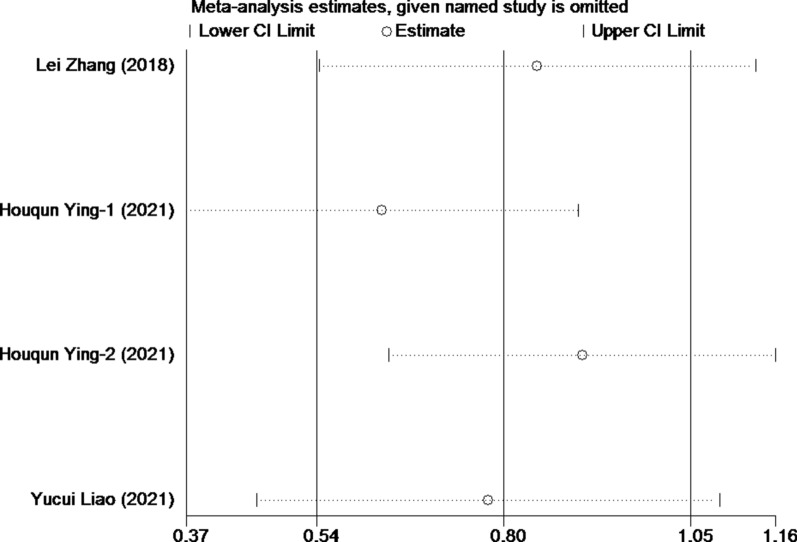
Sensitivity analysis for the association between FPR and RFS. RFS: recurrence-free survival

**Fig. 7:**
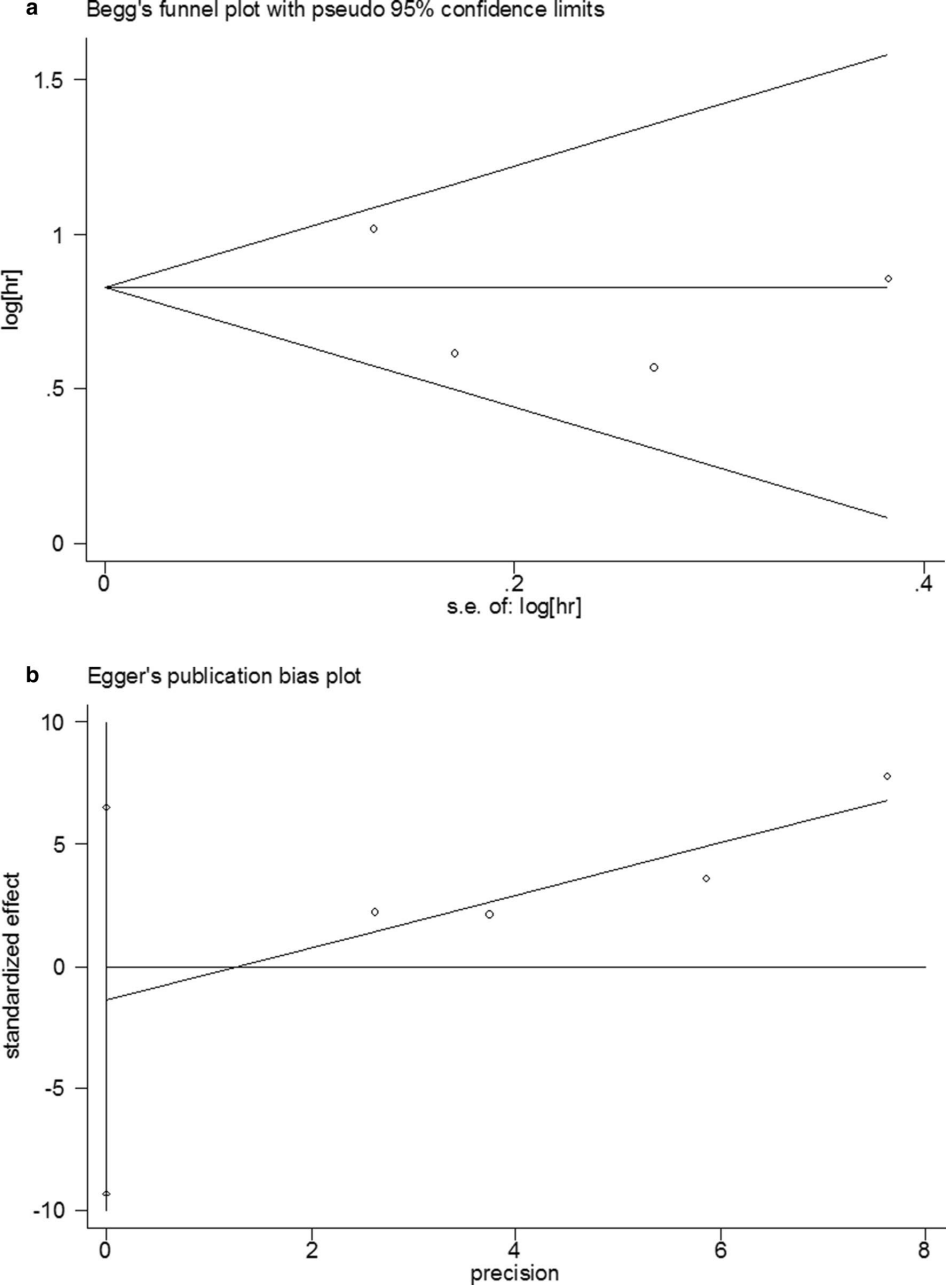
Plots for publication bias test in meta-analysis for recurrence-free survival. **a** Begg’s funnel plot; **b** Egger’s publication bias plot

Four studies involving 1,920 patients reported the association between FPR and postoperative recurrence-free survival in patients with malignant tumors of the digestive system. The comprehensive results showed that a high FPR was related to poor RFS in patients with malignant digestive system tumors (HR = 2.29, 95% CI 1.91–2.76, p < 0.001). In the absence of heterogeneity (I^2^ = 35.3%, P = 0.201), we used a fixed-effect model (Fig. 5). In addition, we performed subgroup analyses based on publication time, sample size, study type, and cancer type. The results showed that FPR was an independent prognostic factor affecting RFS in each subgroup (Table 3) After deleting each study, we recalculated the merger of HR and 95% CI for sensitivity analysis (Fig. 6). The final results suggested that the deletion of any study cohort did not affect the RFS. In other words, the combined results of our meta-analysis were stable. In addition, both Begg's test (p = 1.000) and Egger's test (p = 0.522) indicated no potential publication bias was present in the meta-analysis of RFS (Fig. 7a, b).

**Table 3:** Subgroup Meta-analysis of FPR and RFS

Subgroup	No.of cohorts	No. of patients	Pooled HR (95% CI)	P	Heterogeneity *I*^2^ (%) P_h_	
Altogether	4	1920	2.29(1.91,2.76)	< 0.001	35.3	0.201
Publishing time						
< 2021	1	230	1.77(1.04,2.99)	0.034	NA	NA
≥ 2021	3	1690	2.32(1.73,3.11)	< 0.001	43.6	0.170
Sample capacity						
< 480	2	387	1.94(1.26,2.99)	0.002	0.0	0.534
≥ 480	2	1533	2.38(1.94,2.92)	< 0.001	71.8	0.060
Study designed type						
Retrospective	1	157	2.36(1.12,4.99)	0.025	NA	NA
Prospective	3	1763	2.17(1.59,2.97)	< 0.001	56.8	0.099
Cancer site						
HCC	1	230	1.77(1.04,2.99)	0.034	NA	NA
CRC	3	1690	2.32(1.73,3.11)	< 0.001	43.6	0.170

### Association between FPR and other outcomes

**Fig. 8:**
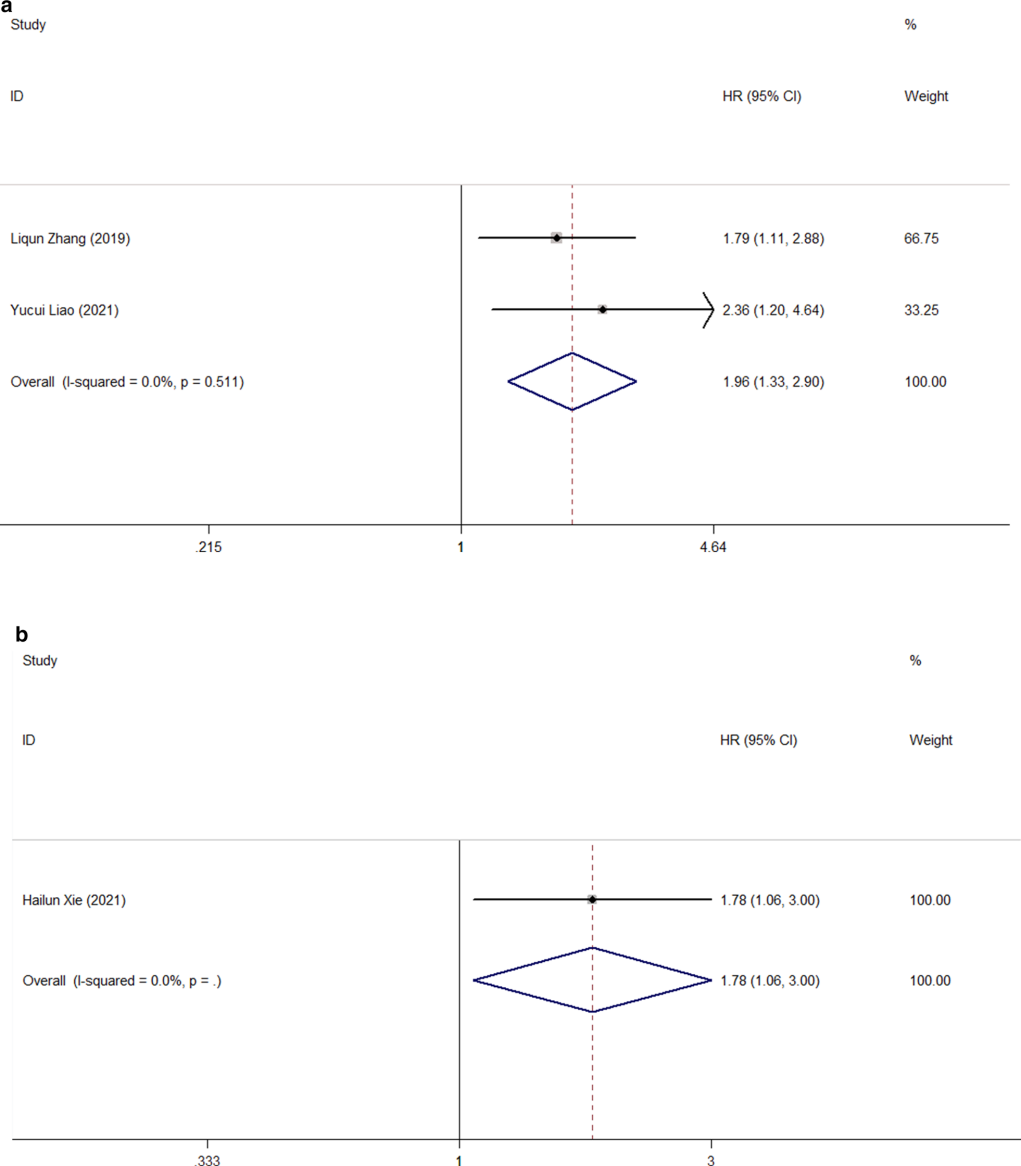

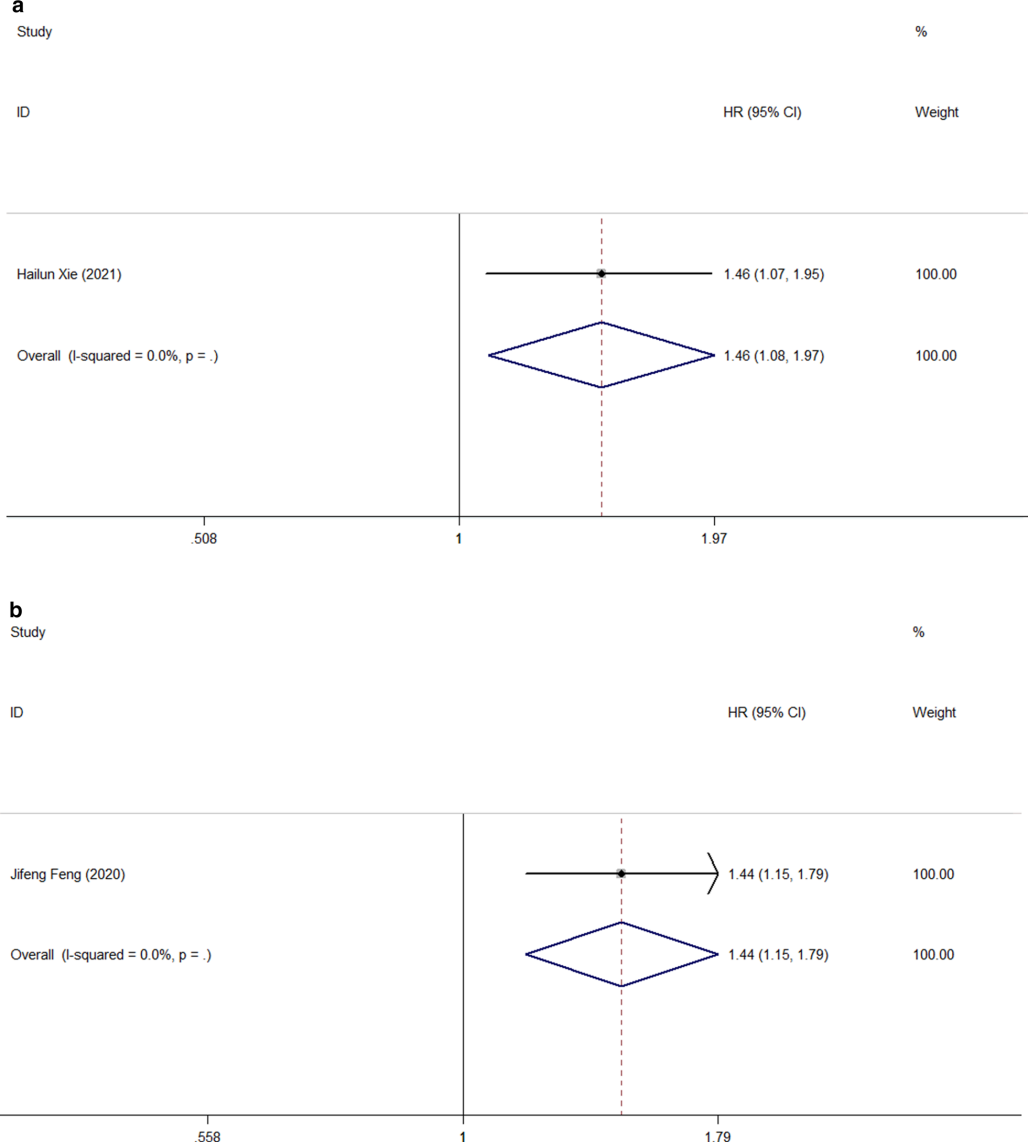
Forest plot for the association between FPR and progression-free survival (**a**)/ complication (**b**)/ disease-free survival (**c**) / cancer-specific survival (**d**)

We also investigated the effects of FPR on the prognosis of complications, PFS, DFS, and CSS in patients with malignant tumors of the digestive system. The prognostic value of FPR for progression-free survival was reported in two cohort studies, including 113 patients (Fig. 8A), using a fixed-effect model due to the absence of heterogeneity. (I^2^ = 0.0%, p = 0.511) The combined results showed that PFS was significantly shorter in patients with high FPR than in patients with low FPR (HR = 1.96, 95%CI: 1.33–2.90, p = 0.001). A study involving 584 patients showed that high FPR was an independent risk factor for complications and disease-free survival (HR = 1.78, 95%CI 1.06–3.00, p = 0.029 and HR = 1.46, 95%CI 1.08–1.97, p = 0.013) (Fig. 8B, C). Similarly, a study involving 372 patients showed that FPR was an independent predictor of cancer-specific survival in patients with malignant tumors of the digestive system (HR = 1.44, 95%CI 1.15–1.79, p = 0.001) (Fig. 8D).

## Discussion

Pre-albumin, also known as transthyretin, is synthesized by the liver and regulates vitamin A and thyroxine synthesis and transportation [8].Given that the half-life of pre-albumin is only about two days, it can reflect minor changes caused by malnutrition and liver insufficiency in a short period [12, 25].Fibrinogen has been reported to be involved in the formation of the inflammatory microenvironment, inflammatory level, angiogenesis, malignant tumor cell proliferation, invasion, and metastasis as an important inflammatory factor [26]. Available meta-analyses suggest that fibrinogen is a prognostic factor for acute myeloid leukemia, venous thromboembolism, cardiovascular disease, and solid tumors [27–30].

Mounting studies have shown that pre-albumin(PA) and fibrinogen regulate the occurrence and development of various tumors, and their levels in peripheral blood are closely related to the patient's survival and sensitivity to therapy [31, 32].As a novel, effective, economical and practical biomarker, the fibrinogen to pre-albumin ratio (FPR) is not only gradually used to evaluate the prognosis of digestive system tumors such as colorectal cancer [20], hepatocellular carcinoma [16] and gastric cancer [13], but also as an index to predict the prognosis of lung [33] and bladder cancer [34]. A study of patients with NSCLC reported that FPR is an independent factor affecting OS in patients with advanced NSCLC [33]. However, most studies on FPR have explored the relationship between FPR and malignant tumors of the digestive system. In other words, PFR may be an important indicator in the field of malignant digestive system tumors research. A study of 230 patients showed that FPR was superior to other biomarkers to independently predict survival of HCC patients and could identify which patients would benefit from adjuvant chemotherapy [16]. Ying et al. [23] suggested that FPR was more effective than other inflammatory markers in predicting recurrence of CRC in stage II-III surgical patients.

Single markers of fibrinogen and pre-albumin are limited and unstable in predicting the prognosis of digestive system tumors [13]. Hailun Xie et al. [20] found that pre-albumin and fibrinogen themselves do not affect postoperative complications and long-term prognosis of patients with colorectal cancer, while FPR can be used as a predictor of postoperative complications and long-term prognosis of colorectal cancer. FPR can not only reflect the level of inflammation in patients, but also indicate the nutritional status of patients [35].Patients with high FPR have reduced nutritional levels and a reduced burden of cancer-related inflammation, which can lead to impaired immune detection and recovery [36]. FPR balances the effects of inflammation and nutrition, and is a comprehensive indicator that reflects a patient's biological status more comprehensively.

The reason why FPR is related to the prognosis of digestive tract tumors is not clear. However, there are several possible mechanisms. Fibrinogen and pre-albumin are liver acute phase positive and negative proteins, respectively [37]. Kris A et al. [38] reported that fibrinogen plays an important role in the occurrence and development of inflammation-dependent cancer. In the process of cancer development, microvascular destruction caused by inflammation results in the accumulation of fibrinogen and other FDPS, which stimulates the secretion of more cytokines and chemokines and further promotes the invasion of cancer [39, 40]. Many studies have shown that fibrinogen increases the metastatic potential of tumor cells. Fibrinogen can act as a bridge between platelets and circulating tumor cells (CTCs), thus promoting platelet adhesion to CTCs [41]. In particular, thrombin can catalyze the conversion of fibrinogen in the circulatory system into a dense fibrin matrix, which then connects with platelets to form a stable skeleton and extracellular matrix around tumor cells to protect them from being killed by immune cells [42, 43]. In addition, fibrinogen can also directly bind to the intercellular adhesion molecule-1 (ICAM-1) of endothelial cells to promote tumor cell migration [44]. In addition, animal experiments showed that tumor cell metastasis was significantly inhibited in fibrinogen deficient mice [45]. Pre-albumin has a shorter half-life than albumin, is more susceptible to dramatic changes in liver function and responds more quickly to nutritional requirements [46]. It has been reported that IL-6 secreted by tumor-related fibroblasts can inhibit pre-albumin and stimulate fibrinogen, resulting in the decrease of pre-albumin and the increase of fibrinogen [37]. In other words, the value of FPR also increases. People with high FPR cues have malnutrition, impaired liver function, hypoproteinemia, and hypercoagulable state, which are usually clinical symptoms of patients with digestive tract tumors. Therefore, the study of FPR will reveal the relationship between cancer-related inflammation, disease and nutrition, indicating the progress of the disease.

Herein, we included 13 studies involving 5116 patients with malignant tumors of the digestive system. Existing evidence indicates that FPR is a sensitive indicator for predicting the prognosis of patients in this population, and patients with a high FPR exhibited worse OS than those with a low FPR. In the meantime, subgroup analysis was also conducted to explore the influence of various factors on our final findings. Although publication time, sample size, cutoff value, source of cutoff value, cancer site, treatment option and type of study varied in different groups, a high FPR was still significantly associated with poor prognosis. We further verified the stability of the meta-analysis by conducting sensitivity analysis and adjusting for censoring. In addition, we further discussed the relationship between FPR and recurrence-free survival in patients. The comprehensive results showed that FPR was an independent predictor of RFS in patients with malignant digestive system tumors. At the same time, subgroup analysis showed that although there were differences in publication time, sample size, research type and cancer system among different subgroups, high FPR still correlated significantly with poor RFS. Sensitivity analysis indicated that our conclusions on RFS were robust, and the funnel chart exhibited no potential publication bias. In addition, we also discussed the relationship between FPR and other prognostic indicators of malignant tumors of the digestive system. A high FPR was associated with adverse clinical outcomes in terms of CSS, DFS, PFS, and complications. In all events, FPR can be considered an important and practical clinical indicator to predict patient prognosis.

To the best of our knowledge, this is the first meta-analysis to comprehensively examine the prognostic value of FPR in patients with malignant tumors of the digestive system. Based on the available evidence, FPR was associated with significant outcomes (OS and RFS) in the present study and correlated with adverse clinical outcome indicators (complication, DFS, PFS and CSS). We also performed a rigorous subgroup analysis and sensitivity analysis to further demonstrate the prognostic significance of FPR in these patients. In addition, the cut-off values of FPR in most of the included studies are mainly concentrated in the range of 18 to 23.1, which provides a certain reference value for clinically determining the critical value of FPR.

However, it should be noted that there are some limitations in our meta-analysis. First of all, all the included studies were conducted in China; accordingly, the drawn conclusions mainly indicate that FPR has certain clinical value in the treatment of patients with malignant tumors of the digestive system in China; accordingly, more large scale studies worldwide are needed to provide sufficient evidence to substantiate the veracity of our findings. Moreover, significant heterogeneity was present in our meta-analysis, which may have been caused by the small number of included studies and samples. Finally, due to the limited number of included studies, the cutoff value of FPR in CSS, DFS, PFS and complications remains to be explored.

## Conclusion

According to this meta-analysis, high FPR is significantly correlated with poor prognosis, which can be harnessed clinically as an indicator to predict the prognosis of patients with malignant tumors of the digestive system. However, large multicenter studies worldwide are still needed to substantiate our findings.

## Data Availability

Please contact author for data requests.

## Abbreviations

FPR: Fibrinogen to pre-albumin ratio
95% CI: 95% Confidence interval
OS: Overall survival
CSS: Cancer-specific survival
DFS: Disease-free survival
RFS: Recurrence-free survival
PFS: Progression-free survival
